# Optimal radiation dose for patients with one to three lymph node positive breast cancer following breast-conserving surgery and anthracycline plus taxane-based chemotherapy: A retrospective multicenter analysis (KROG 1418)

**DOI:** 10.18632/oncotarget.12882

**Published:** 2016-10-25

**Authors:** Haeyoung Kim, Won Park, Jeong Il Yu, Doo Ho Choi, Seung Jae Huh, Yeon-Joo Kim, Eun Sook Lee, Keun Seok Lee, Han-Sung Kang, In Hae Park, Kyung Hwan Shin, Kyubo Kim, Kyung Ran Park, Yong Bae Kim, Sung Ja Ahn, Jong Hoon Lee, Jin Hee Kim, Mison Chun, Hyung-Sik Lee, Jung Soo Kim, Jong-Young Lee

**Affiliations:** ^1^ Department of Radiation Oncology, Hallym University Dongtan Sacred Heart Hospital, Hwaseong, Gyeonggi, South Korea; ^2^ Department of Radiation Oncology, Samsung Medical Center, Sungkyunkwan University School of Medicine, Seoul, South Korea; ^3^ Center for Breast Cancer, Research Institute and Hospital, National Cancer Center, Goyang, South Korea; ^4^ Department of Radiation Oncology, Seoul National University College of Medicine, Seoul, South Korea; ^5^ Department of Radiation Oncology, Ewha Womans University Mokdong Hospital, Ewha Womans University School of Medicine, Seoul, South Korea; ^6^ Department of Radiation Oncology, Yonsei Cancer Center, Yonsei University College of Medicine, Seoul, South Korea; ^7^ Department of Radiation Oncology, Chonnam National University Medical School, Gwangju, South Korea; ^8^ Department of Radiation Oncology, St. Vincent's Hospital, The Catholic University of Korea College of Medicine, Suwon, South Korea; ^9^ Department of Radiation Oncology, Dongsan Medical Center, Keimyung University School of Medicine, Daegu, South Korea; ^10^ Department of Radiation Oncology, Ajou University School of Medicine, Suwon, Gyeonggi, South Korea; ^11^ Department of Radiation Oncology, Dong-A University Hospital, Dong-A University School of Medicine, Busan, South Korea; ^12^ Department of Radiation Oncology, Chonbuk National University Medical School, Jeonju, Jeollabuk, South Korea; ^13^ Department of Radiation Oncology, Wonju Severance Christian Hospital, Wonju, Kangwon, South Korea

**Keywords:** breast neoplasms, radiotherapy, dose-response relationship, prognosis

## Abstract

**Background and Purpose:**

This study was performed to determine optimal radiation dose in pN1 breast cancer patients who received breast conserving surgery (BCS) and anthracycline plus taxane (AT)-based chemotherapy.

**Materials and Methods:**

Retrospective chart reviews were performed in 1,147 patients who were treated between January 2006 and December 2010. The impact of radiation dose on treatment outcomes was evaluated.

**Results:**

Median follow-up time was 66 months. The 5-year rate of disease-free survival (DFS) was 93.2%. Larger tumor size (> 20 mm), positive lymphovascular invasion, high histologic grade, and high ratio of positive nodes (> 0.1) were significantly associated with inferior DFS. By using the 4 factors related to DFS, patients were categorized into high-risk (with ≥ 3 factors) and low-risk (with < 3 factors) groups. In the high-risk group, higher radiation dose (> 60.3 Gy_EQD2_) was significantly associated with better DFS than the lower dose (≤ 60.3 Gy_EQD2_). However, the radiation dose did not impact DFS in the low-risk group.

**Conclusions:**

Dosing of radiation affects the outcome of post-BCS radiotherapy in pN1 breast cancer. Doses of over 60.3 Gy_EQD2_ were associated with better outcome in the high-risk patients.

## INTRODUCTION

Radiotherapy after breast-conserving surgery (BCS) reduces the rates of recurrence and death from breast cancer [1]. A meta-analysis showed that post-BCS radiotherapy reduces the rate of recurrence by 15.7% and decreases the risk of breast cancer death by 3.8% [1]. However, even if the benefit of post-BCS radiotherapy has been proven, the optimal dose for radiotherapy remains uncertain.

According to the latest treatment guidelines, whole breast irradiation (WBI) with or without a boost should be administered in patients treated with BCS [2–5]. However, optimal dosing for radiation has not been undetermined. Even though there have been a number of trials that have evaluated prognostic impact of different radiotherapeutic regimens on tumor control, most of the patients included in those trials were treated with less effective systemic therapy than the current standard chemotherapeutic regimen available for breast cancer [6–8].

Addition of chemotherapeutic agents to four cycles of anthracycline and cyclophosphamide (AC) has been shown to be more effective than the AC regimen alone [9]. Moreover, adjuvant endocrine therapy and anti-human epidermal growth factor receptor-2 (HER2) treatment decreased the risk of breast cancer recurrence in subgroups of patients [10, 11]. Given the effectiveness of systemic treatments, absolute gains in tumor control by radiotherapy might be diminished, thus requiring further modifications of radiotherapeutic regimens in the background of patients receiving effective systemic treatments. In addition, adjustment of radiation dose based on the recurrence risk for each patient allows individualizing radiotherapy for breast cancer treatment.

In the current study, we categorized pN1 breast cancer patients into risk groups depending on the recurrence risk and analyzed the impact of radiation dose on disease control in each risk group. Through this analysis, we aimed to provide an optimal regimen for post-BCS radiotherapy in pN1 breast cancer patients who received systemic treatments including anthracycline plus taxane-based (AT) chemotherapy.

## RESULTS

Among the 1,147 patients, 1,139 (99.3%) had a T1 or T2 tumor while 8 (0.7%) had a T3 tumor. Details of patient's and tumor characteristics are shown in Table 1. Median follow-up time of the patients was 66 months (range, 3-112 months). A total of 86 (7.5%) patients were found to have disease recurrence. As the first failure, recurrence in local, regional, distant, and simultaneous loco-regional and distant sites were detected in 9 (0.8%), 7 (0.6%), 53 (4.6%), and 17 (1.5%) patients, respectively. Contralateral breast cancer was detected in 8 (0.7%) patients during the course of follow-up after radiotherapy. The 5-year rate of OS, DFS, LRRFS, and DMFS of the patients were 98.6%, 93.2%, 97.3% and 94.3%, respectively.

**Table 1:** Patient's characteristics

Characteristics		No. of patients (%)
Age	Median 47 (range, 21-76)	
	≤ 40	231 (20.1)
	> 40	916 (79.9)
		
Pathology	IDC	1078 (93.9)
	Non-IDC	69 (6.1)
		
Tumor size	Median 20 mm (range, 0.1-75 mm)	
	≤ 20 mm	588 (51.3)
	> 20 mm	559 (48.7)
		
Number of tumor	Single	954 (83.1)
	Multiple	193(16.9)
		
Resection margin	Negative	1138(99.2)
	Positive	9 (0.8)
		
LVI	Negative	453 (39.4)
	Positive	694 (60.6)
		
HG	1,2	713 (62.2)
	3	434 (37.8)
		
Molecular subtype	Luminal A	595 (51.8)
	Luminal B	180 (15.7)
	Luminal-HER2	117 (10.2)
	HER2 enriched	69 (6.1)
	Triple negative	186 (16.2)
		
No. of positive nodes	1	668 (58.2)
	2	303 (26.4)
	3	174 (15.4)
		
Ratio of positive nodes*	≤ 0.1	693 (60.4)
	> 0.1	454 (39.6)
		

Abbreviations: IDC = invasive ductal carcinoma, LVI = lymphovascular invasion, HG = histologic grade, HER-2 = human epidermal growth factor receptor2.

* Ratio of positive lymph nodes of the total dissected lymph nodes.

**Figure 1:**
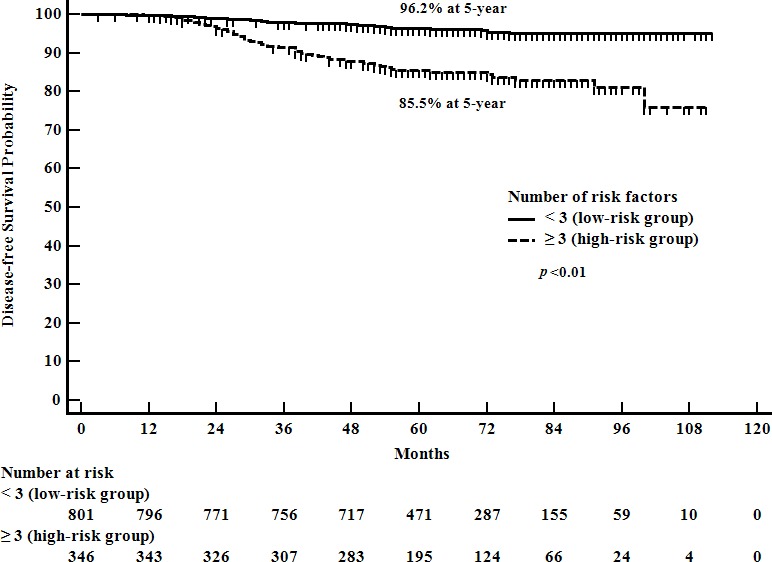
Disease-free survival according to the number of risk factors

Larger tumor size (> 20 mm), positive LVI, high HG (grade 3), and high ratio of positive nodes (> 0.1) were significantly related to inferior DFS in univariate and multivariate analyses (Table 2). The patients with luminal A type tumor had a better DFS than the patients with non-luminal A type cancer in the univariate analysis. However, the statistical significance of the molecular subtype for DFS was not found in the multivariate analysis. By using the aforementioned four prognostic factors, we categorized the patients into two groups as high-and low risk groups. Patients with three or more prognostic factors were classified as high-risk, while patients with less than three prognostic factors were categorized as low-risk. The 5-year DFS rate was significantly different according to the risk groups (96.2% *vs*. 85.5%, *p* < 0.01, Figure 1).

**Table 2:** Prognostic factors for disease-free survival

		5-year DFS (%)	*P*-value		
Characteristics			Univariate	Multivariate	HR (95% CI)
**Age**	≤ 40	90.8	0.06	-	
	> 40	93.5			
					
**Tumor size**	≤ 20 mm	96.5	<0.01	<0.01	2.4 (1.5-3.9)
	> 20 mm	89.3			
					
**Number of tumor**	Single	93.0	0.69	-	
	Multiple	92.7			
					
**Resection margin**	Negative	92.9	0.67	-	
	Positive	100.0			
					
**LVI**	Negative	96.6	<0.01	<0.01	2.0 (1.2-3.5)
	Positive	91.0			
					
**HG**	1,2	96.2	<0.01	<0.01	2.6 (1.6-3.9)
	3	87.9			
					
**Molecular subtype**	Luminal A	96.3	<0.01	-	
	Non-luminal A	89.5			
					
					
**No. of (+) node**	1	94.3	0.05	-	
	2 & 3	91.1			
					
Ratio of (+) node*	≤ 0.1	94.7	<0.01	<0.01	1.8 (1.2-2.8)
	> 0.1	90.4			

Abbreviations: DFS = disease-free survival, HR = hazard ratio, CI = confidence interval, LVI = lymphovascular invasion, HG = histologic grade.

**Figure 2:**
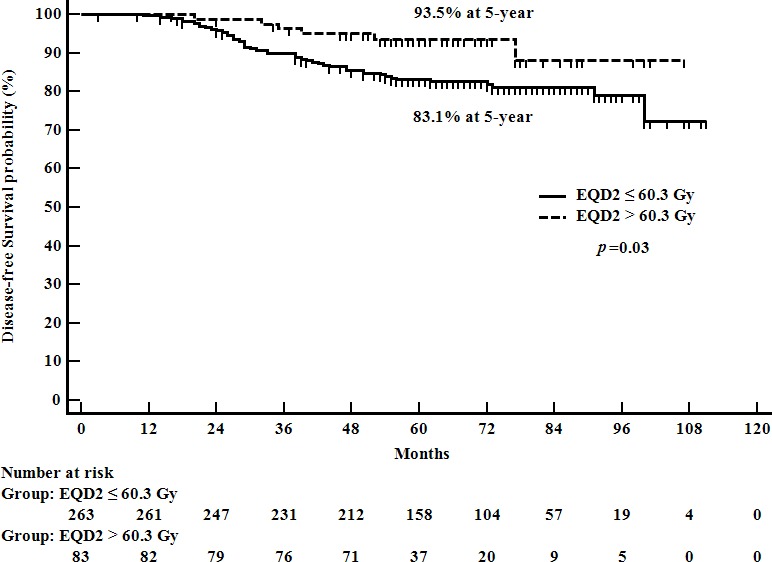
Disease-free survival according to radiation therapy dose received among the high-risk group patients

To evaluate the impact of radiation dose on treatment results, we analyzed the patient survivals according to the total radiation dose received. Patients who received higher radiation dose than 60.3 Gy_EQD2_ showed better DFS than the patients treated with lower than or equal to 60.3 Gy_EQD2_ (92.1% *vs*. 95.3% at 5-year, *p* = 0.02). The impact of total radiation dose on DFS was statistically significant in the high-risk group, while it was not significant in the low-risk group. In the high-risk group, the patients treated with higher doses (> 60.3 Gy_EQD2_) showed significantly better DFS and LRRFS rates than the patients who received lower doses (≤ 60.3 Gy_EQD2_) (Table 3, Figure 2). Among the high-risk patients, the characteristics between the higher dose (> 60.3 Gy_EQD2_) and lower dose (≤ 60.3 Gy_EQD2_) groups were not statistically different (Supplementary Table 1).

**Table 3:** Impact of radiation dose on survivals according to risk groups

		All patients (*N* = 1147)			Patients in low-risk group (*N* = 801)			Patients in high-risk group (*N* = 346)		
Survival	Dose (EQD2)	*N*.	5-year (%)	*p*-value	*N*	5-year (%)	*p*-value	*N*.	5-year (%)	*p*-value
DFS	≤ 60.3 Gy	828	92.1	0.02	565	96.3	0.70	263	83.1	0.03
	> 60.3 Gy	319	95.3		236	96.0		83	93.5	
LRRFS	≤ 60.3 Gy	828	96.6	0.01	565	98.4	0.27	263	92.8	0.03
	> 60.3 Gy	319	99.0		236	99.1		83	98.7	
DMFS	≤ 60.3 Gy	828	93.6	0.12	565	97.0	0.78	263	86.3	0.06
	> 60.3 Gy	319	95.7		236	96.0		83	94.7	
OS	≤ 60.3 Gy	828	98.3	0.17	565	99.5	0.99	263	96.0	0.07
	> 60.3 Gy	319	99.0		236	98.6		83	100.0	

Abbreviations: DFS = disease-free survival, LRRFS = loco-regional recurrence-free survival, DMFS = distant metastasis-free survival, OS = overall survival, EQD2 = biologically equivalent dose in 2 Gy fractions.

## DISCUSSION

The result of the current analysis suggests that dosing of radiation significantly affects the outcome of post-BCS radiotherapy in patients with pN1 breast cancer who received AT-based chemotherapy. We developed a prognostic model for predicting DFS and identified patients at high-risk of disease recurrence. There is a significant relationship between radiation dose and tumor control. Patients treated with a higher total radiation dose than 60.3 Gy_EQD2_ obtained better DFS than the patients who received a radiation dose lower than or equal to 60.3 Gy_EQD2_. The impact of the radiation dose on DFS was pronounced in the high-risk group while it was not significant in the low-risk group.

We found that tumor size, LVI, HG, and ratio of positive lymph node were significant factors for predicting DFS. A prognostic model using the four risk factors effectively predicted patient's prognosis. Among the significant factors, the ratio of positive nodes was a better predictor of prognosis than the number of positive lymph node according to the multivariate analysis in the current study. This finding is consistent with the previous studies [12, 13]. Several recent studies have reported that the disease outcome was significantly associated with molecular subtype of the tumor [14, 15]. The current study observed the significant impact of molecular subtype on DFS in the univariate analysis. However, the statistical significance was lost when the multivariate analysis was performed. As previously presented in a study [16], it is likely that predictive power of the molecular subtype is less than that of pathologic features in early-stage breast cancer.

It has been shown that applying AT-based chemotherapy reduces disease recurrence and breast cancer mortality more effectively than prescribing anthracycline-based regimen alone in patients with early breast cancer [9]. Also, adding taxane to anthracycline improves tumor control not only for distant organs but also for locoregional sites [17]. Moreover, endocrine therapy and anti-HER2 treatment reduces locoregional recurrence by about 50% when they are properly conducted according to molecular subtype [18–20]. In the present study, all patients were treated with AT-based chemotherapy. Nearly all patients with hormone-responsive breast cancer received adjuvant endocrine treatment and more than half of the patients with HER2-amplified tumor were treated with anti-HER2 therapy. Such thorough systemic treatments appear to have contributed to favorable locoregional control in the current analysis as we found the 5-year locoregional recurrence rate to be 2.7%. However, the high-risk patients who had three or more risk factors had significantly poorer DFS than the low-risk patients, suggesting that further intensification of treatment is required for the high-risk patients.

To find an optimal dose of radiotherapy, we evaluated the influence of total radiation dose on a patient's DFS. EQD2 of over 60.3 Gy was significantly associated with better DFS for all patients. The impact of high radiation dose on disease outcome was different in each risk group. Among the patients with low-risk disease, there was no difference in survival according to the radiation dose. Regardless of radiation dose, however, favorable disease control was observed in the low-risk patients. On the contrary, in the high-risk group, patients treated with higher doses achieved better survival rates than those receiving lower radiation doses. Statistically superior outcomes by the high dose were observed for DFS and LRRFS in the high-risk patients. It is assumed that in high-risk patients, possible remnant disease after BCS cannot be successfully eradicated with the current AT-based systemic treatments and radiotherapy lower than 60.3 Gy_EQD2_.

A few studies have evaluated dose-response relationship in post-BCS radiotherapy through randomized trials of WBI with or without tumor bed boost. The studies included patients who had mostly pN0 disease and received no chemotherapy or had non-taxane based chemotherapy. In these trials, boost radiation of 10-16 Gy was administered after WBI of 50 Gy with a daily dose of 2-2.5 Gy. The addition of tumor bed boost resulted in better local control and prolonged DFS more than WBI alone [6, 21, 22]. Likewise, we found that there was a positive effect of high dose radiation on tumor control in patients with pN1 breast cancer. Unlike the abovementioned boost trials, all patients were treated with AT-based systemic treatments and only high-risk patients benefited from the high dose radiation in the current study. Therefore, it is conceivable that even with effective systemic treatments, high dose radiation is necessary for selected patients receiving post-BCS radiotherapy.

In the present study, boost radiotherapy was performed in about 98% of the patients and different dosing schemes of WBI and tumor bed boost were applied. Therefore, differences in radiation dose across the patients were caused by variations in total radiation dose to whole breast and tumor bed. Hence, it is difficult to settle on an adequate individual dose value for the whole breast and tumor bed in the present analysis. Rather, our study determined that a total radiation dose of over 60.3 Gy_EQD2_ was optimal for achieving fair outcomes. Considering that the total dose prescribed in the patients allocated to the boost group was 62.5-66 Gy_EQD2_ in the aforementioned boost trials [6, 21, 22], the dose of 60.3 Gy_EQD2_ recommended in our study is rather low. It is likely that the radiation dose necessary for disease control could have been reduced by integrating effective systemic treatments.

There are limitations in the current study. Firstly, we assessed the doses of radiotherapy based on retrospective chart reviews from 12 different hospitals. Each hospital used different techniques and protocols of radiotherapy. Since the radiation dose to target and surrounding normal organs is affected by techniques of radiotherapy [23], the treatment outcomes might have affected by the techniques used. Secondly, we could not evaluate radiation-related toxicity as not all the participant hospitals could provide the information. There have been studies reporting risk of skin toxicity by increased radiation dose in patients with breast cancer [6, 21]. Likewise, it is possible that the patients treated with a dose over 60.3 GyEQD2 had increased risk of skin toxicity in our study. In order to determine the benefit of increased dose of post-BCS radiotherapy, assessing treatment-related toxicity is necessary in the future study.

In conclusion, we identified the high-risk group in the patients with pN1 breast cancer who were treated with post-BCS radiotherapy and AT-based chemotherapy. Higher total dose of over 60.3 Gy_EQD2_ was closely associated with favorable outcome, particularly in high-risk patients. Based on these results, we expect to individualize post-BCS radiotherapy according to the patient's risk of disease recurrence.

## MATERIALS AND METHODS

In the current retrospective multicenter cohort study, we included 1,147 patients with pN1 breast cancer treated at the radiation oncology department in 12 hospitals in Korea between January 2006 and December 2010. The inclusion criteria were pN1 breast cancer patients receiving BCS and AT-based adjuvant chemotherapy, completion of planned radiotherapy, and having information on pathologic features of the tumor including hormone receptor status. The exclusion criteria were patients treated with neoadjuvant chemotherapy, chemotherapy other than AT-based regimen, incomplete follow-ups, or previous history of breast radiotherapy.

Retrospective chart reviews were performed to collect pathologic features of tumor such as tumor size, number of positive lymph nodes, histologic grade (HG), presence of lymphovascular invasion (LVI), resection margin (RM), and expression status of estrogen receptor (ER), progesterone receptor (PR), and HER2. The ER and PR positivity were defined as having any positive nuclear staining, and HER2 positivity was defined as having an immunohistochemistry (IHC) score of 3+ or an IHC score 2+ along with a positive fluorescent *in situ* hybridization (FISH) or a positive chromogenic *in situ* hybridization (CISH) for HER2 gene amplification. Molecular subtypes of breast cancer were categorized as follows: ER+ or PR+, HER2-, and HG 1 or 2 (i.e. luminal A); ER+ or PR+, HER2-, and HG3 (i.e. luminal B); ER+ or PR+, HER2+ (i.e. luminal HER2); ER-, PR-, and HER2+ (i.e. HER2 enriched); ER-, PR-, and HER2- (i.e. triple negative).

All patients received axillary lymph node dissection (ALND). The median number of examined lymph nodes was 16 (range, 1-48). As for chemotherapy, doxorubicin and cyclophosphamide (AC) or epirubicin and cyclophosphamide (EC) followed by paclitaxel or docetaxel (T) were prescribed in all patients. Among the patients with hormone-responsive tumors, 861 (96.5%) patients were treated with adjuvant endocrine therapy. For patients with HER2+ tumors, anti-HER2 treatment was given to 116 (62.3%) patients.

All patients had a WBI with a total dose of 45.0-60.4 Gy at a 1.8-3.0 Gy per fraction. Boost irradiation was administered to 1,127 (98.3%) patients with a total dose of 4.0-19.8 Gy at a 1.8-3.5 Gy per fraction. Conventionally fractionated WBI with a daily dose of 1.8-2.0 Gy was performed in 1,079 (94.1%), while hypofractionated WBI with a total dose of 51.0 Gy in 17 fractions at a 3.0 Gy per fraction was delivered to 68 (5.9%) patients. A total of 364 (31.7%) patients had supraclavicular lymph node radiotherapy (SCN RT) with a total dose of 40.0-60.0 Gy in 20-30 fractionations. All patients underwent computed tomography (CT) simulation. For WBI, opposed tangential beams with conventional two-dimensional (2D) radiotherapy was used in 1,067 (93.0%) patients while tangential field intensity-modulated radiotherapy (IMRT) was performed on 80 (7.0%) patients. Tumor bed boost was delivered using an electron beam or three-dimensional conformal photon beams per institutional policy. Radiotherapy was delivered after completion of chemotherapy in 1,003 (87.4%), sequentially administered with chemotherapy as a sandwich approach in 117 (10.2%), or given before chemotherapy in 27 (2.4%) patients. To evaluate equivalent radiation doses in different radiotherapy schedules, the biologically equivalent dose in 2 Gy fractions (EQD2) of the total radiation dose including WBI and boost was calculated, using the linear quadratic model with α/β = 10 for tumor.

After radiotherapy, patients were followed up according to each institution's protocol. Typically, history taking and physical examination were performed every three to six months for the first three years, and every six months for years four and five, and followed annually thereafter. A mammogram exam was performed annually on all patients. Bilateral breast ultrasonography (US) or magnetic resonance image, Chest X-ray or CT, abdomen US or CT, bone scan, or whole-body fluorine 18-fluorodeoxyglucose positron emission tomography (PET)/CT were performed to patients according to clinical situations.

The overall survival (OS), disease-free survival (DFS), locoregional recurrence-free survival (LRRFS), and distant metastasis-free survival (DMFS) were defined as interval from surgery to death, cancer recurrence, loco-regional recurrence, and distant metastasis, respectively. The study analyzed prognostic factors affecting patient's DFS. Among the factors, the number of tumors, RM, LVI, HG, and hormone receptor status were considered as binary variables. The patient's age, tumor size, number of positive nodes, ratio of positive nodes, and EQD2 were analyzed as continuous variables. An optimal cut-off of the continuous variables was defined using analysis of the area under the curve (AUC) of receiver operating characteristics (ROCs). The value for which sensitivity and specificity were the highest has been chosen as the optimal cut-off point for each variable. The survival probability was estimated using the Kaplan-Meier method and the Log-rank test was used to compare survivals between the groups with different variables. To determine the independent prognostic factors for survival, Cox regression analysis with stepwise selection was used. Statistical significance was calculated at the 95% confidence interval (*p*-value < 0.05) and all the analyses were performed with the Statistical Package for the Social Sciences (SPSS) version 22.0 (IBM SPSS Statistics for Windows; IBM Corp, Armonk, NY, USA). The present study was approved by the Korean Radiation Oncology Group (KROG) along with the Institutional Review Board of each hospital that participated in the study.

## SUPPLEMENTARY MATERIAL


