# Supplementary material for: Optimal radiation dose for patients with one to three lymph node positive breast cancer following breast-conserving surgery and anthracycline plus taxane-based chemotherapy: A retrospective multicenter analysis (KROG 1418)
Source: Oncotarget. 2016 Oct 25;8(1):1796–804. doi: 10.18632/oncotarget.12882 (PMC5352099; doi:10.18632/oncotarget.12882)
Supplement: Supplementary file 1 [file oncotarget-08-1796-s001.pdf]

Optimal radiation dose for patients with one to three lymph node positive breast cancer following breast-conserving surgery and anthracycline plus taxane-based chemotherapy: A retrospective multicenter analysis (KROG 1418)

Supplementary Material

Supplementary Table 1. Comparison of characteristics between the patients with higher EQD2 (> 60.3 Gy) and lower EQD2 (≤ 60.3 Gy) among the high-risk patients

**Characteristics**		**EQD2** ≤ 60.3Gy (N=263)	**EQD2** > 60.3 Gy (N=83)	*P*-value
**Age**	≤ 40	54 (20.5)	21 (25.3)	0.35
	> 40	209 (79.5)	62 (74.7)	
**Tumor size**	≤ 20 mm	39 (14.8)	7 (8.4)	0.14
	> 20 mm	224 (85.2)	76 (91.6)	
**Number of tumor**	Single	231 (87.3)	70 (84.3)	0.41
	Multiple	32 (12.7)	13 (15.7)	
**Resection margin**	Negative	261 (99.2)	81 (97.6)	0.24
	Positive	2 (0.8)	2 (2.4)	
**LVI**	Negative	17 (5.3)	7 (8.4)	0.56
	Positive	246 (94.7)	76 (91.6)	
**HG**	1,2	77 (29.3)	21 (25.3)	0.48
	3	186 (70.7)	62 (74.7)	
**Molecular subtype**	Luminal A	196 (74.5)	68 (81.9)	0.18
	Non-luminal A	67 (25.5)	15 (18.1)	
**Ratio of (+) LN**	≤ 0.1	71 (26.9)	23 (27.7)	0.89
	> 0.1	192 (73.1)	60 (72.3)	
**SCN RT**	No	146 (55.5)	47 (56.6)	0.85
	Yes	117 (44.5)	36 (43.4)	
**Hormonal Tx***	No	6 (2.3)	4 (4.8)	0.17
in ER+ and/or PR+	Yes	177 (97.7)	48 (95.2)	
**Anti-HER2†**	No	16 (32.0)	7 (30.4)	0.89
in HER2+	Yes	34 (68.0)	16 (69.6)	

Abbreviations: EQD2 = biologically equivalent dose in 2 Gy fractions.

*in ER+ and/or PR+, n=235; †in HER2+ n=73.
